# Strategies for recruitment in general practice settings: the iSOLVE fall prevention pragmatic cluster randomised controlled trial

**DOI:** 10.1186/s12874-019-0869-7

**Published:** 2019-12-11

**Authors:** Amy C. W. Tan, Lindy Clemson, Lynette Mackenzie, Catherine Sherrington, Chris Roberts, Anne Tiedemann, Constance D. Pond, Fiona White, Judy M. Simpson

**Affiliations:** 1 Faculty of Health Sciences, The University of Sydney, Lidcombe, New South Wales Australia; 2 Sydney School of Public Health, The University of Sydney, Camperdown, New South Wales Australia; 3 Sydney Medical School, The University of Sydney, Camperdown, New South Wales Australia; 4 School of Medicine & Public Health (General Practice), University of Newcastle, Newcastle, New South Wales Australia

**Keywords:** General practice, General practitioners, Primary care, Fall prevention, Randomised controlled trial, Cluster randomisation, Recruitment

## Abstract

**Background:**

Falls are common among older people, and General Practitioners (GPs) could play an important role in implementing strategies to manage fall risk. Despite this, fall prevention is not a routine activity in general practice settings. The iSOLVE cluster randomised controlled trial aimed to evaluate implementation of a fall prevention decision tool in general practice. This paper sought to describe the strategies used and reflect on the enablers and barriers relevant to successful recruitment of general practices, GPs and their patients.

**Methods:**

Recruitment was conducted within the geographical area of a Primary Health Network in Northern Sydney, Australia. General practices and GPs were engaged via online surveys, mailed invitations to participate, educational workshops, practitioner networks and promotional practice visits. Patients 65 years or older were recruited via mailed invitations, incorporating the practice letterhead and the name(s) of participating GP(s). Observations of recruitment strategies, results and enabling factors were recorded in field notes as descriptive and narrative data, and analysed using mixed-methods.

**Results:**

It took 19 months to complete recruitment of 27 general practices, 75 GPs and 560 patients. The multiple strategies used to engage general practices and GPs were collectively useful in reaching the targeted sample size. Practice visits were valuable in engaging GPs and staff, establishing interest in fall prevention and commitment to the trial. A mix of small, medium and large practices were recruited. While some were recruited as a whole-practice, other practices had few or half of the number of GPs recruited. The importance of preventing falls in older patients, simplicity of research design, provision of resources and logistic facilitation of patient recruitment appealed to GPs. Recruitment of older patients was successfully achieved by mailed invitations which was a strategy that was familiar to practice staff and patients. Patient response rates were above the expected 10% for most practices. Many practices (*n* = 17) achieved the targeted number of 20 or more patients.

**Conclusions:**

Recruitment in general practice settings can be successfully achieved through multiple recruitment strategies, effective communication and rapport building, ensuring research topic and design suit general practice needs, and using familiar communication strategies to engage patients.

**Trial registration:**

The trial was prospectively registered on 29 April 2015 with the Australian New Zealand Clinical Trial Registry www.anzctr.org.au (trial ID: ACTRN12615000401550).

## Background

Falls are common in older age; one in three people aged 65 years and over living in the community fall each year.[1] Causes of falls are multifactorial, and effective evidence-based interventions include balance exercises, medication review, and home safety adaptations [1]. Given the multifactorial nature, general practitioners (GPs) as the primary healthcare provider could potentially play an important role in engaging patients holistically [2, 3]. The iSOLVE (Integrated Solutions for Sustainable Fall Prevention) project aimed to integrate a fall prevention decision tool within general practices located in a Primary Health Network geographical area in Sydney, Australia [4]. The project included a pragmatic cluster randomised controlled trial (CRCT) to test a clinical multifactorial fall prevention intervention whilst simultaneously implementing the intervention in real practice setting, also referred as a type 2 hybrid effectiveness-implementation study in the protocol paper [4]. This required recruitment of general practices, GPs and their patients [4].

Fall prevention is not a common activity in Australian general practice that is focused on chronic disease management and acute treatments in older patients [5]. Therefore, GPs’ beliefs and responsiveness to fall prevention is not known, and their recruitment into the iSOLVE CRCT was anticipated to be challenging if GPs did not see the relevance of the research topic [6–14]. An additional barrier for the iSOLVE CRCT was the trial intervention aiming for GPs to change practice by routinising a comprehensive multifactorial intervention for all older patients 65 years and over, [4, 15] balanced with higher-priority clinical and management demands of the daily operations of general practice [8, 12, 14, 16–18]. Further, there is no clear model for implementation of fall prevention in general practices in Australia and internationally to guide the iSOLVE CRCT. For instance, the Cochrane Review (2012) [1] on fall prevention in the community indicated only four of 159 international trials involved real-practice GPs in conducting interventions to prevent falls [19–22], whereas other fall prevention trials had only used GPs and/or GP practices for referral to trial intervention(s), case finding, medical clearance or inspection of records. Despite these issues, example recruitment strategies used by other studies were able to guide iSOLVE recruitment, including using champions or peer recruitment [8, 11, 16, 17, 23–25], associating with an organisation or university [10, 13, 17, 26], road shows or face-to-face promotions, [9, 23, 27] mailed invitations [10, 28], and cold-calling [9, 27].

Delays and difficulty in achieving patient recruitment targets have also been reported, which may lead to an extended recruitment period, funding issues or inadequate statistical power [7, 29–33]. A review of 34 general practice randomised trials in the United Kingdom reported that only 30% of trials recruited within the planned duration, and that five times as many trials recruited on schedule when the researchers (not the GP) were responsible for gaining patient consent.[7] Other authors reported clinician-led recruitment barriers such as: inability to commit time [17, 29, 32, 33], difficulty communicating trial information, [14, 30, 32] and patient selection bias [32]. The level of patient interest can also impact on recruitment targets, [29, 31, 32] and to some extent multiple strategies can boost patient recruitment including using practice records, screening in waiting room, and advertisements in practices [7, 31]. While several community-based fall prevention trials [1] showed that patient recruitment could be achieved in general practice, the challenge remains for the iSOLVE CRCT to achieve patient recruitment targets within each general practice cluster in addition to GP recruitment targets.

Our aims were to describe and assess the strategies used to recruit practices, GPs within the practices and their patients and, secondly, to reflect on enablers and barriers relevant to recruitment in the context of the iSOLVE CRCT. Findings reported in this paper are considered novel given the research gap on fall prevention in general practice and are anticipated to inform recruitment strategies for future cluster intervention trials. While there have been other intervention trials conducted in general practice settings [8, 12–14, 16, 17, 24–26], this is the first multifactorial fall prevention CRCT in Australian general practice and one of the few general practice intervention-based trials focusing on fall outcomes internationally [19–22].

## Methods

### Aim, design and setting of the study

The aims of this paper were to describe and assess the strategies used to recruit practices, GPs within the practices and their patients and, secondly, to reflect on enablers and barriers relevant to recruitment in the context of the iSOLVE CRCT. The CRCT was nested within a type 2 hybrid effectiveness-implementation study [4].

Participants were recruited from the Northern Sydney area, Australia. The iSOLVE CRCT targeted 28 GP practices and 560 patients (20 patients per practice), as per sample size estimated in the protocol paper [4]. Recruitment of practices and GPs were undertaken by a full-time project coordinator (AT1) who also conducted training of GPs allocated to the intervention group[4]. Cluster randomisation was undertaken at the practice level; AT1 was blinded to the group allocation sequence when recruiting GPs. Once GP recruitment and patient lists at the practice was finalised, the practice was then randomised, at which point AT1 was unblinded in order to proceed with coordinating the practices during the trial including recruiting patients and implementing interventions. Recruitment of patients was undertaken by a full-time research assistant (FW), who was always blinded to the group allocation of practices.

### Recruitment of general practices and GPs

The iSOLVE CRCT was conducted in partnership with an Australian government primary health organisation, the Northern Sydney Medicare Local (NSML), which subsequently transitioned into the Sydney North Primary Health Network (SNPHN) in July 2015. While this affected the NSML’s and SNPHN’s capacity to engage their local general practices in a timely manner, the advantage was the doubling of the geographical study area from 150 to 284 general practices for potential recruitment. All general practices and GPs within the NSML and SNPHN were invited to participate. Information was distributed through a mailed invitation-to-participate, verbal presentations at practices and workshops/forums, online GP fall prevention survey, iSOLVE connections with health professionals, and various newsletters, to maximise reach to general practices and GPs (Table 1).

**Table 1:** Strategies to initiate contact with general practices and to communicate trial information to GPs

Communication strategies	*Active communication instigated and followed up by project coordinator (AT1) with the intention of engaging and recruiting GPs*	*Passive communication via third party with the intention of increasing awareness and interest for the trial*	*Unique cases supporting usefulness of strategy*
Electronic (2–3 sentences paragraph in an email or newsletter)	• **Online survey**: GP fall prevention survey (to GPs and practice staff) distributed using emails in NSML database in mid-2015, as part of the iSOLVE larger project [4], which led to an expression of interest form on completion of the survey. • **Email invitation**: o Invitation-to-participate emailed by one of the research investigators to GP-academics at the Department of GP at the University of Sydney. o Invitation-to-participate emailed to GP attendees who participated at an NSML aged care forum in 2014, where fall prevention guidelines and trial information were presented amongst other topics.	• **iSOLVE webpage**: A specific iSOLVE webpage on NSML and SNPHN website displayed information about the trial. • **Clinical audit activity:** iSOLVE-led fall prevention clinical audit activity accredited for GP continuing professional development promoted on SNPHN education page. • **Newsletters**: o Trial information distributed via NSML and SNPHN newsletters (approximately bi-annually). o Newsletter distributed by the School of Public Health at the University of Sydney to promote the medication management workshop and the RACGP clinical audit activity, where trial information was provided to attendees.	• A practice did not respond to repeated invitations to participate, but the practice nurse discovered the research on the SNPHN website and engaged the practice GPs to contact iSOLVE.
Written (2-page double-sided flyer of trial summary on the first page and images of resources on the second page for mailout and distribution)	• **Mailouts**: Two mailouts of promotion flyer and expression of interest form to individual GPs using addresses from NSML database, supplemented by publicly available web-based business directories. • **Medication management workshop**: Faxes to general practices (2015, 2016) and mailout to individual GPs (2016) to promote two iSOLVE-led “medication management for preventing falls” workshop in 2015 and 2016, where written and oral trial information was provided to attendees.	• **Word of mouth within iSOLVE network:** o Written trial information provided to allied health professionals who attended iSOLVE-led education workshops as part of the iSOLVE larger project [4], and other NSML or SNPHN education events. o Rarely, patients who were keen for their GPs to participate (after learning about the trial through their peers) were provided with a flyer to show their GP, with a follow-up personalised invitation-to-participate addressed to the GP from project coordinator (AT1).	• A practice nurse had expressed interest through an NSML network but could not engage GPs to participate, however the GPs later responded to the mailed invitation to participate. • Two GPs expressed interest through the medication workshop, despite them being mailed the invitation to participate or having previously completed the online survey.
Oral (5–15 min presentations depending on time allocated)	• **Word of mouth within iSOLVE network:** Potentially interested general practices identified through the iSOLVE network such as recruited GPs, advisory group members, allied health professionals, and NSML or SNPHN staff. • **Presentation at GP practices:** Trial information presented at practices that organised group education visits for their GPs and staff.	• **Word of mouth within iSOLVE network:** Oral trial information provided to allied health professionals who attended iSOLVE-led education workshops as part of the iSOLVE larger project [4]. • **University Department of GP presentation:** Trial information presented at a research showcase organised by the Department of General Practice at the University of Sydney in 2016.	• Two practices were interested at the point of receiving the mailed invitation to participate but had not responded and would not have been recruited without the practice presentations. • Two practices did not respond to mailed invitation but responded when contacted after being identified through the iSOLVE network.

SNPHN is the study area, Sydney North Primary Health Network; NSML is the former study area, Northern Sydney Medicare Local

Once informal contact was established with a GP or practice, a practice visit was organised to provide information and to obtain consent from interested GPs. The practice visit emphasised the significance of fall prevention, the aim of the trial for GPs to implement fall prevention management with their patients [4], randomisation process (practice level), and the benefits of the trial intervention, such as accredited GP activities relating to fall prevention in older people (educational visiting and clinical audit), clinical resources, and a list of service providers for referral. AUD$100 was offered to thank each participating GP for their contribution to the research. The simplicity of the research logistics for the GP was also highlighted: completion of a survey at baseline, 3-months and 12-months, and screening of their patient list, as well as implementation of fall prevention management with older patients as part of their routine clinical practice for the intervention. GPs were also informed that they would receive the education and resource intervention at baseline if their practice was randomised to the intervention group or at the end of 12-months if their practice was randomised to the control group. For practices with multiple GPs, once one GP had consented to participate, others were provided written and/or oral information, and invited to participate. Practice-wide participation was encouraged [4]. ‘Rolling’ recruitment and randomisation was used to minimise delays, and for stages of the research to occur at each practice at their convenience, independent of other practices.

### Recruitment of general practice patients

Using the practice database, the project coordinator (AT1) liaised with either the practice manager, nurse, receptionist or GP to generate a list of patients 65 years and over, who were seen by participating GPs. To recruit the targeted 20 patients per practice, the list aimed to contain approximately 200 patients across all recruited GPs in the practice, allowing for an expected 10% participant response rate, based on iSOLVE investigators’ expert knowledge and previous trial experiences [34]. In larger practices, patient lists were capped by applying additional filters such as patients seen within a certain period (1 month-2 years) or the random selection of a certain percentage of patients (1–2%). Recruited GPs were asked to scan their respective patient list to exclude patients who met the exclusion criteria, such as those not living independently in the community, receiving active treatment for a serious illness (e.g. chemotherapy), diagnosed with dementia, or in palliative care. Patients with significant mental health issues or language barriers, and those who were not their regular patients (e.g. only seen once) were excluded at the GPs’ discretion.

At the practice, the project coordinator used a standard letter template and printed letters addressed to individual patients, incorporating the practice letterhead and the name(s) of participating GP(s), which were posted out through the university mailing system. The posting of invitation letters was a communication strategy that was familiar to many practice staff and patients. Interested patients used either reply-paid mail, telephone or email to contact the research assistant (FW), who was blinded to the practice group randomisation conducted prior to recruiting the practice’s first patient [4]. The research assistant screened the patients for their eligibility (i.e. worried about falling or had at least one fall in the past year) and suitability to participate in the CRCT. The research assistant visited the patient at a convenient place (e.g. home) to obtain consent and collect baseline data. Patients did not need to travel to the university to participate but were required to discuss fall prevention with their respective GP as part of the intervention (at baseline for intervention practices, or at 12-months for control practices). Patients were informed that they might incur out-of-pocket expenses for GP appointments and any fall prevention services referred by their GP as part of the intervention. The research assistant also collected monthly fall calendars via reply-post or telephone over a period of 12 months for each recruited patient.

### Data analysis

Mixed methods were used to collect quantitative and qualitative data simultaneously to complement contextualisation of the strategies and results of the recruitment [35, 36]. The project coordinator (AT1) noted strategies, numbers and characteristics of each general practice and GP encountered, as well as field notes reflecting on the point of contact, recruitment process, and communications to identify factors that enabled or hindered GPs and practice staff’s involvement in the CRCT. Interactions which occurred between the research assistant (FW) and patients were reflected orally to the project team and unique details were noted.

Quantitative data were analysed descriptively using frequency tables to explore comparisons of recruitment rates, results of recruitment strategies, recruitment numbers and practice, GP and patient characteristics. Reflective analysis to identify common enablers and barriers was applied to narrative data in the field notes [37] Regular meetings were conducted with the lead investigators (LC, LM) which enabled reflection on actions or events that influenced the CRCT. To enhance validity of narrative data, a series of qualitative interviews (transcribed verbatim), with one focusing on recruitment and engagement, were conducted by LM with AT1 which enabled consolidation and triangulation of data. Both the meetings and interviews also enabled ‘thinking-aloud’, reflective recall, critical mapping and peer debriefing to further strengthen the analysis of the field notes [37]. Identified findings were cross-examined and validated by co-authors (LC, LM, FW).

## Results

### Timeline

**Fig. 1:**
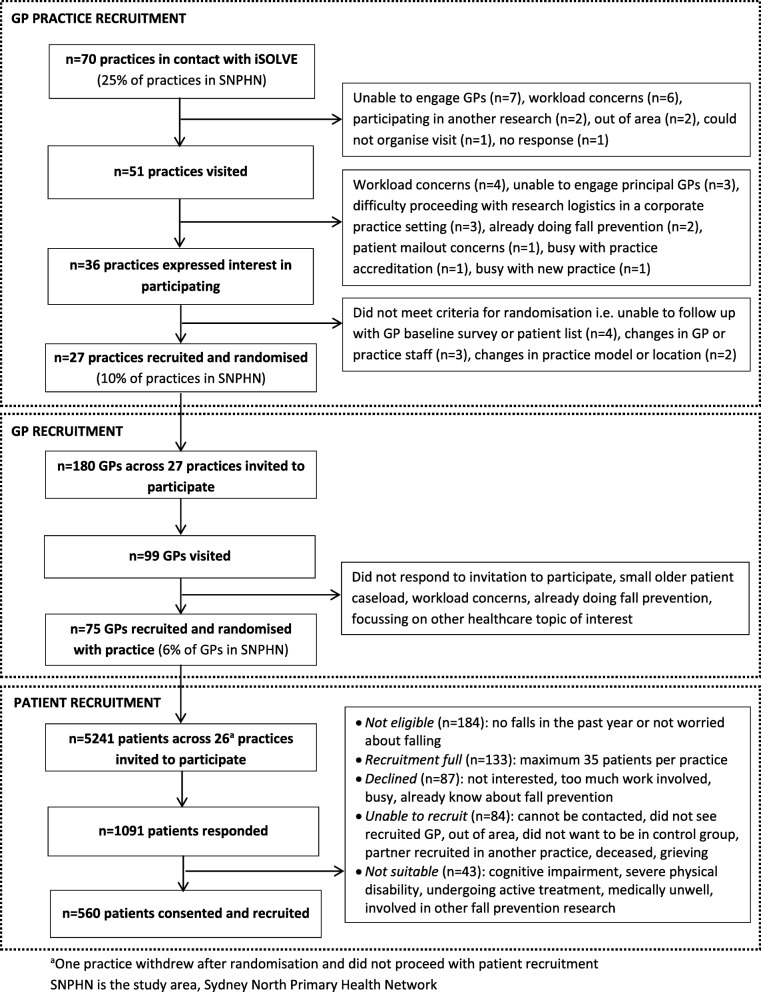
Recruitment of practices, GPs and patients flowchart, and reasons for decline

Twenty-seven GP practices and 75 GPs were recruited between June 2015 and November 2016 (Fig. 1). Initial engagement continued beyond the targeted 28 practices to adjust for loss of GP practices prior to randomisation for various reasons (Fig. 1). Five-hundred-sixty patients were recruited between June 2015 and January 2017 (Fig. 1).

On average, it took 1.5 months (range: 0.5–3.5 months) for a practice visit to be organised from the point of contact. It took an average of 3 months (range: 0.5–9 months) to start patient recruitment after the GP(s) completed baseline surveys and screened the patient list, and the practice was randomised. The main reasons for delays included being busy with prioritised tasks such as practice accreditation, and difficulty in aligning multiple GPs (e.g. on leave, busy) in medium and large practices. Patient recruitment took an average of 2 months (range: 1–5 months) to complete. No association was identified between time taken, number of GPs recruited and practice size.

### Strategies to initiate contact with general practices and to communicate trial information to GPs

A total of 188 GPs (16% of 1162 GPs in study area in 2016) and 12 practice staff across 70 practices (25% of 284 practices in study area in 2016) were contacted by the iSOLVE team. Table 2 describes the response and recruitment results for each strategy used to engage practices. The multiple strategies were collectively useful in reaching our targeted number, as evidenced by case studies in Table 1. Overall, strategies that enabled higher responses and recruitment numbers appeared to be those that involved direct contact with GPs, such as individualised invitations, online GP surveys, and face-to-face presentations. GPs and practice staff reported being overwhelmed by other emails or postal mails which contributed to the overall low response rates for similar recruitment strategies. Despite the costs involved, practice visits increased awareness of fall prevention and the iSOLVE CRCT, and were successful in engaging GPs who may not have otherwise participated. The eventual recruitment rate was limited by the difficulty with engaging the appropriate GPs (e.g. principal GP or GPs with a caseload of older patients) and/or making contact at the appropriate time (e.g. accreditation period, systemic changes at the practice, appropriate GPs not present at time of visit). Contact via the iSOLVE network and educational events (e.g. workshops, seminars, and the offer of a clinical audit activity) may have complemented other strategies to influence engagement.

**Table 2:** General practice and GP recruitment results (from highest to lowest number of practices recruited)

Recruitment strategy	Mailout to GPs		Online survey (GPs, practice staff)	Presentation at GP practices	Contact via word of mouth within iSOLVE professional network	Medication management workshop		iSOLVE webpage	University Department of GP presentation	Clinical audit activity	Newsletters and email invitations
Baseline number	432 GPs (2015)	890 GPs (2016)	1400 emails, 41 responses	18 practices	Unknown	5 GPs (2015)	11 GPs (2016)	Unknown	30–50 attendees	Unknown	Unknown
Informal contact n = 70 practices	8	8	13	18	10 (allied health *n* = 4, SNPHN n = 4, GP *n* = 2)	4	4	1	1	3	0
Visited n = 51 practices	7	7	7	18	6	2	2	1	1	0	0
Expressed interest n = 36 practices	3	6	7	12	3	2	1	1	1	0	0
Total recruited n = 27 practices (% contacted)	**9** **(56%)**		**6** **(46%)**	**5** **(28%)**	**3** **(30%)**	**2** **(25%)**		**1** **(100%)**	**1** **(100%)**	**0** **(0%)**	**0** **(0%)**
Total GPs recruited n = 75 GPs	18		16	18	7	4		6	6	0	0
Relative cost	$$ (postage)		Nil^a^	$$$ (catering^b^)	Nil	Nil^a^		Nil	Nil	Nil^a^	Nil
Advantages enabling recruitment	Identify GPs with interest in fall prevention		Identify GPs with interest in fall prevention	Face-to-face promotion	Identify GPs with interest in fall prevention	Identify GPs with interest in fall prevention, face-to-face promotion		Increase awareness for iSOLVE	Face-to-face promotion, increase awareness for iSOLVE	Incentive for participation	Increase awareness for iSOLVE
Disadvantages and barriers to recruitment	Can get lost amongst other letters		Can get lost amongst other emails	Costly, relevant GPs not available at time of visit, not available for every practice	Difficult to go through practice staff as the gate keeper of enquiries if the GP(s) did not directly express interest	GPs interested in fall prevention education but not able to participate in research		Reach unknown, competing with other promotional efforts	Audience profile varied and included non-GPs, out of area or non-practising GPs	GPs not able or interested to participate in research	Can get lost amongst emails, competing with other promotional efforts

^a^No additional costs incurred as the activities were conducted as part of the iSOLVE larger project [4]

^b^Catering costs for GP practice presentation were higher because catering was considered for all available GPs and staff at the practice rather than being limited to GPs and staff who expressed interest through other strategies

### Strategies to engage and recruit general practices and GPs

Twenty-seven GP practices and 75 GPs were recruited. A wide range of sizes of GP practices engaged with iSOLVE, and there was a mix of individual GPs and practice-wide participation (Table 3). The CRCT recruited more medium-sized practices which seemed to be due to the availability of a key person (mainly practice manager or nurse) who would take on a facilitator role (Table 3) to assist with collating GP surveys, collating patient lists, organising visits, and communicating between the project coordinator and the GPs. Medium-sized practices were easier to engage more quickly than large practices.

**Table 3:** Types of general practices, general practice characteristics, GP characteristics, and number of patients recruited

Practice size represented by number of GPs in each practice^a,b^	Small practices		Medium-sized practices		Large practices		Total
	Solo GP	2–3 GPs	4–6 GPs	7–10 GPs	11–20 GPs	> 20 GPs	
General practice characteristics	***Number of practices***						
Practices recruited	5	2	8	8	3	1	**27**
Person in facilitator role							
GP	3	1	-	2	-	-	**6**
Practice manager	-	1	5	4	3	-	**13**
Nurse	2	-	2	2	-	1	**7**
Main receptionist	-	-	1	-	-	-	**1**
GP recruitment status							
All/most GPs recruited	5	1	3	3	-	-	**12**
Half of practice recruited	-	-	5	3	1	-	**9**
Only one or a few GPs recruited	-	1	-	2	2	1	**6**
GP characteristics	***Number of GPs***						
GPs recruited	5	3	25	29	11	2	**75**
Gender^b^							
Male	4	1	9	11	3	2	**30**
Female	1	2	16	18	8	-	**45**
Years in practice as a GP							
< 5 years	-	-	3	1	-	-	**4**
5–10 years	1	-	2	3	1	-	**7**
> 10 years	4	3	20	25	10	2	**64**
Percentage patients ≥65 years							
1–20%	1	2	5	6	5	1	**20**
21–40%	4	1	11	10	6	1	**33**
41–60%	-	-	6	10	-	-	**16**
61–80%	-	-	3	3	-	-	**6**
Patient recruitment number	***Number of patients***						
Number of patient mailouts	383	602	1881	1746^c^	494	135	**5241**
Number of patients responded *Response rate (% mailed)*	64 *17%*	83 *14%*	388 *21%*	401 *23%*	126 *26%*	29 *21%*	**1091** ***21%***
Target patient number to recruit (20 patients per practice)	100	40	160	160^c^	60	20	**540**
Number of patients recruited *Recruited rate (% mailed)*	35 *9%*	39 *6%*	207 *11%*	189 *11%*	70 *14%*	20 *15%*	**560** ***11%***

^a^At baseline. Medium-sized practices tended to employ regular practice staff to assist with daily practice administration compared to small practices; large practices tended to employ even more practice staff and had multiple co-located services

^b^When comparing with unpublished SNPHN (study area) data (2017), GP practices recruited were under-representative of solo and small GP practices (2–5 GPs) (55% iSOLVE, 75% SNPHN) and over-representative of medium-large GP practices (over 5 GPs) (44% iSOLVE, 24% SNPHN). The female:male ratio of GPs in our study (60:40) is similar to SNPHN data (58:42)

^c^Patient recruitment was conducted with 26 GP practices as 1 practice (3 GPs) withdrew after randomisation and did not proceed with patient recruitment

The in-practice face-to-face information session prior to obtaining informed consent was valuable in providing designated time with minimal interruptions to build rapport with GPs, facilitator person and practice staff. This session also enabled understanding of crucial practice-based enablers for conducting the CRCT such as adapting to other priorities at the practice and working with the practice’s routine system. Strategies such as information provision over the phone, “passing the message” by GPs, practice nurse or staff, or leaving written information for absent GPs did not generate further interest with only three additional GPs recruited by their peers.

For most recruited practices, at least one principal GP participated or approval was obtained from the principal GP and/or management. A significant barrier to practices proceeding with the trial was difficulty in engaging the principal GP and/or the practice manager, particularly when the practice was focused on major management activity or structural change (Fig. 1). In practices where the facilitator role was not clear, AT1 engaged a person to undertake the role, but many of these practices did not eventually proceed with the CRCT. It was also possible that the lack of overall interest in some practices (where only a few GPs expressed interest) might have discouraged the practice manager or person undertaking the facilitator role to involve the practice.

### Enablers, barriers and GPs’ incentives for participation

Many GPs recognised the importance of preventing falls due to their experience with older patients, someone close to them having fallen, or their own fall experience. They understood the consequences of falls in terms of injuries, reduced confidence and the impact of these things on the ability to stay independent. Most recruited GPs had more than 10 years’ experience (Table 3) and they felt that this topic was important for enhancing their own practice and improving their patients’ health, even though some admitted that they rarely responded to research initiatives. However, interest in fall prevention did not guarantee participation in the CRCT, because: 1) a focus on other health topics of interest, 2) those with a high caseload of older patients were either too busy or were semi-retired, 3) some reported insufficient older patients on their caseload, and/or 4) some GPs believed they were already implementing fall prevention in their practice.

Most questions from GPs related to the research objectives, research team and funding, research design, and intervention implementation. The practice visits by someone close to the research (i.e. project coordinator), who also conducted the CRCT intervention, enabled prompt responses to questions to minimise delays in decisions to participate. Workload was a major concern, and recruited GPs were appreciative of the simplicity of the research requirements. Some GPs specifically identified the iSOLVE intervention components [4], such as the clinical decision tool and list of service providers, as valuable in addressing gaps in fall prevention in GP practice settings.

Some GPs indicated that being involved in a university-based initiative appealed to them, due to the quality and rigour of the research. The provision of activities accredited for continuing professional development was viewed as valuable, although not essential as many GPs did not eventually participate in the optional clinical audit activity. A token of AUD$100 per GP was offered to acknowledge their time with the research; this was considered a bonus but did not appeal to those GPs who were not interested in fall prevention or research involvement.

### Recruitment results for older patients

Patients were recruited from 26 practices as one practice withdrew after randomisation. Twelve practices generated lists of 200 patients or more as per the initial research protocol; eight practices generated 100–199 patients and six practices generated less than 100 patients. This was dependent on the recruited GPs’ caseload of older patients rather than practice size or number of GPs recruited (Table 3). Some GPs had underestimated their caseload when asked to estimate the percentage of patients seen who are 65 years or older (Table 3).

The higher mailout numbers resulted in a higher number of responses and patients eventually recruited. The average response rate was 21% (range: 7–38%). Of the three practices with the lowest response rate (7, 10 and 12%), two were new solo practices (less than 6 months old) at the time of recruitment and one practice (size: 2–3 GPs) included inactive patients on their list. This was unsurprising as almost all patients who responded were generally regular or ‘loyal’ patients of their GP. Most patients were respectful of their GP’s recommendation to participate in the project, while also interested in preventing falls.

Five-hundred-sixty patients (180 males and 380 females) were recruited. The average age was 78 years old (range: 65–95). With an average recruitment rate of 11% (range: 5–24%), 17 practices achieved the targeted 20–35 patients recruited (capped to minimise variability between practices), four practices recruited 10–19 patients, and five practices recruited less than 10 patients. A second mailout was offered to an initial six practices with less than ten patients recruited (four solo practices and two medium/large practices), and only one solo practice declined the second mailout. Among the five practices, the second mailout did not markedly increase responses or recruitment for four practices where the letter was resent to the same list of patients and therefore did not serve its purpose as a reminder letter, but did increase responses for one practice which doubled its mailout number to include different patients.

The research assistant observed an increased number of male patients through male GPs (a third of recruited participants), the inclusion of ethnic groups due to some GPs’ cultural background (e.g. Middle Eastern, East/South-East Asian, European), and reasonable diversity in socio-economic status due to the spread of recruited practices across metropolitan Northern Sydney (Socio-economic Indexes for Areas (SEIFA) scores ranging from 1010 to 1164, with NSW state and national SEIFA ranges being 779–1164 and 554–1196 respectively) [38].

## Discussion

The iSOLVE CRCT was successful in reaching the targeted number of participants and to engage enough practices to allow for any attrition within sample size estimated in the protocol paper [4]. The strength of this CRCT lies in the relatively larger numbers of GP practices and GPs recruited, compared to the few existing fall prevention trials [19–22]. In addition, this paper contextualises strategies in achieving recruitment targets in a trial of this size and intervention nature requiring GPs to change practice and to provide fall prevention interventions.^4^ Whilst we had no control over GPs’ expression of interest, our broad recruitment of GPs from various general practice settings, and the resulting diversity in patients recruited, should strengthen sample representativeness. The recruitment success also indicates the relevance of the falls prevention topic in general practice and the value of the iSOLVE CRCT intervention, both of which are recognised as a key element in other trials [7–14, 39].

While recruitment of practices and GPs was boosted by using multiple strategies, as highlighted in other studies [9–11, 17, 28], findings in this paper further described the breadth of strategies needed to accommodate different general practice settings or GPs, and to enable wider reach, rather than focusing on which strategy is superior. In line with some studies, mailing was cost-effective [9, 27, 28], and in-practice presentations were expensive and time-consuming but valuable [23, 27, 39]. Professional connections did not encourage GP participation for this CRCT, in contrast to other general practice studies [10, 11, 13, 16, 17, 24–26], which could have shifted the focus from ‘initiative-active’ practices known to professional networks. Possible explanations for difficulty engaging through these networks were the competing demands from the range of research activities in the study area [27], and the complex intervention that required multi-level ‘buy-in’ [10] including the practices and patients rather than just the individual GP. Overall, we concur with other authors [10, 27], that recruitment requires a significant investment of: time, labour and resources to implement multiple recruitment strategies. We underestimated these in our project and the duration to complete recruitment was longer than the grant research plan of one year. For example, we underestimated the time taken to engage practices to participate, and the time taken to recruit multiple GPs at one practice before being able to randomise and then recruit patients. We also underestimated the need for a variety of strategies to maximise reach, which can be both labour-intensive and resource-heavy. This needs to be taken into account when designing and justifying trial budgets to enable multiple approaches in reaching and engaging potential participants.

When engaging practices, we identified rapport building and relationships with both participants (i.e. GP) and non-participants (i.e. practice staff) within the GP practice by an integral member of the research team (AT1) as an important factor to overcome some barriers. Practice variability can challenge communication [10, 14, 16], and in the iSOLVE CRCT rapport building was specifically enhanced by face-to-face practice visits which enabled individualised interactions. The use of a single recruiter to recruit practices for a trial of this size also enabled adaptation of communication approaches due to increased experience interacting with the GPs and staff throughout the recruitment process. Additionally, GP understanding of the CRCT design varied as found in other studies [8, 12, 32], and clear communication in the iSOLVE CRCT was facilitated by the recruiter who was familiar with the research. Some studies highlighted the presence of a ‘champion’ practice staff as a driver [12, 16, 26, 39], and the iSOLVE recruitment further emphasised the facilitator role of the ‘champion’ staff (usually a non-GP) as a significant deciding factor in the practice proceeding with the trial.

Some studies have reported the need to minimise research burden for GP-participants [8, 13, 24, 32], but this can be challenging if the trial requires the GP to conduct the intervention, unlike one study employing additional intervention clinical staff [13]. Removing GPs from patient recruitment responsibilities have simplified their involvement in the trial, as identified by other studies [11, 13, 14, 24, 27, 32],and enabled GPs to focus on implementing the iSOLVE intervention in their clinical practice. In addition, we also kept a degree of flexibility to ease trial participation: 1) allowing a lengthy participation timeline according to the practice’s needs (within reasonable research timeframe), a difficulty highlighted by other studies [16, 17, 27]; 2) the research stage at each practice was independent of other practices, a challenge reported in another study [12]; and 3) not enforcing the targeted patient number in small practices. A final strategy was offering practice enhancement incentives, as reported in other studies [8, 10, 17, 23], although payment incentives were not perceived to be as crucial as the relevance and ease of the research [8, 11].

The number of patient mailouts and response rates were variable, but the success of the patient recruitment in this study can be attributed to several enablers. The ‘letter-from-GP’ approach was convenient and familiar to patients, as used in other studies [1, 11, 13], while minimising recruitment bias. The CRCT’s simple eligibility criteria (65 years and over) for generating letters was effective, without requiring the screening of patient medical records for specific criteria in contrast to other clinical studies [11, 17, 24]. Due to the success of patient recruitment in many practices, the iSOLVE CRCT did not utilise other patient recruitment strategies, such as recruiting from the waiting room [11, 24, 26], which might be unfavourable given the potential increase in workload for receptionists and the lack of privacy for small general practices.

### Limitations

We did not evaluate the costs of the recruitment initiatives. However, the mixed method approach added in-depth insights into the iSOLVE CRCT recruitment process. There may be potential bias in the data source from the perspective of first person observation data (AT1), but peer-checking and triangulation strengthened the findings. Findings may be limited to the context of cluster randomised trials and fall prevention as the research topic, however, we believe that they add to the literature by providing a comprehensive account of the contexts of the recruitment process in general practice, and the enablers reported are balanced with more commonly reported barriers in the literature. We do not have the full quantitative data on ethnicity and socioeconomic status, however, have included our observation in the results section acknowledging the lack of broad ethnic and socioeconomic coverage in trials as highlighted in a systematic review [11].

## Conclusion

Our study shows that general practice and GP recruitment can be successfully achieved with multiple strategies, efforts to ensure the research design suits the needs of general practice, as well as striving for effective communication and rapport. Patient recruitment targets can also be achieved by ensuring suitable approaches are used. However, estimated time frames and subsequent resource demands need to be carefully planned when determining budgets. While we acknowledge that many complex intervention trials vary in purpose and methodology, the findings presented in this paper contextualise the necessary steps to facilitate trial recruitment. This should be applicable to a broad number of primary care trials of similar size and nature, in Australia and internationally.

## Supplementary information

**Additional file 1.** CONSORT 2010 checklist of information to include when reporting a cluster randomised trial and Extension of CONSORT for abstracts to reports of cluster randomised trials.

## Data Availability

All data supporting the findings of this study are available within the article. CONSORT 2010 checklist of information to include when reporting a cluster randomised trial is included as Additional file 1.

## Abbreviations

CRCT: cluster randomised controlled trial
GP: general practitioner
iSOLVE: Integrated Solutions for Sustainable Fall Prevention
NSML: Northern Sydney Medicare Local
SNPHN: Sydney North Primary Health Network
